# Repeat Kidney Biopsies in Anti-Neutrophil Cytoplasmic Autoantibody-Associated Vasculitis: Clinical and Histologic Progression

**DOI:** 10.1016/j.ekir.2023.07.021

**Published:** 2023-07-29

**Authors:** Faten Aqeel, Lillian Xu, Duvuru Geetha

**Affiliations:** 1 Department of Internal Medicine, Division of Nephrology, Johns Hopkins University School of Medicine, Baltimore, Maryland, USA; 2 Department of Medicine, Johns Hopkins University School of Medicine, Baltimore, Maryland, USA

**Keywords:** ANCA, biopsy, kidney, vasculitis

## Introduction

Antineutrophilic cytoplasmic antibody (ANCA)-associated vasculitis is a multisystemic autoimmune disease that affects small and medium blood vessels. ANCA-associated vasculitis includes 3 distinct clinical phenotypes, namely granulomatosis with polyangiitis, microscopic polyangiitis, and eosinophilic granulomatosis with polyangiitis. Circulating autoantibodies to neutrophil myeloperoxidase and/or proteinase 3 are present in majority of patients with ANCA-associated vasculitis, whereas 10% of patients can be ANCA-negative. Despite recent advancements in induction and maintenance therapies, relapse remains common. A challenging aspect of ANCA-associated vasculitis management is the assessment of disease activity, particularly in cases of renal involvement. The presence of hematuria and proteinuria at diagnosis can reflect kidney involvement. Studies have shown that despite achieving clinical remission, hematuria is seen in up to 40% of patients and proteinuria can persist in up to 45% of patients.^1^^,^^2^ Although a kidney biopsy at initial presentation is often performed to establish a diagnosis and predict prognosis, only a few studies looked at the utility of repeat kidney biopsies at follow-up.^3^, ^4^, ^5^, ^6^, ^7^ It has been demonstrated that the clinician’s impression of disease activity is incorrect in 25% of cases, supporting the utility of a follow-up kidney biopsy to guide management.^6^

Given the paucity of data on the indications, clinical, and histologic progression of repeat kidney biopsies in ANCA glomerulonephritis (ANCA-GN), we sought to study those parameters on initial kidney biopsy (KB1) and repeat for-cause kidney biopsies (KB2) in patients with ANCA-GN. Supplementary References and the Supplementary Methods are included in the Supplementary Material.

## Results

Twenty-nine patients were included in the study. The median age was 73 years (interquartile range 61–81). The mean (SD) time from KB1 to KB2 was 42 (±3.6) months and the median (interquartile range) time from KB1 to KB2 was 34 (12–59) months. The mean follow-up was 11 years. Of the patients, 55% were females and the majority were Caucasian (83%). Fifty-nine percent were myeloperoxidase-ANCA positive and 31% were proteinase-3-ANCA positive. At the time of KB2, ANCA status was persistently positive (28%), newly positive (31%), unknown (21%), and negative (17%). The most common indication for a repeat kidney biopsy was an elevation in serum creatinine (*n* = 16), followed by proteinuria (*n* = 14), and new or persistent hematuria (*n* = 10) equally. The details of KB2 indications are presented in Supplementary Table S1. Of the 29 patients, 11 (38%) had active pauci-immune crescentic GN and the remaining patients had an alternate pathology in KB2. Median (interquartile range) serum creatinine (mg/dl) was 2 (1.6–4.5) in KB1 and 1.9 (1.2–3.8) in KB2 (*P* = 0.322). Median (interquartile range) serum estimated glomerular filtration rate (ml/min per 1.73 m^2^) was 32 (13–46.5) in KB1 and 33 (15–60) in KB2 (*P* = 0.142) (Table 1). Among the 16 patients with elevation in serum creatinine, 8 had active GN on KB2. Among the 14 patients with proteinuria, only 4 had active GN on KB2. Among the 10 patients with persistent hematuria, only 2 had evidence of active GN on KB2. Among 10 patients with new onset hematuria, 7 had evidence of active GN on KB2.

**Table 1:** Patient characteristics, demographics, kidney biopsy findings, and treatment course (*n* = 29)

Baseline Characteristics	Value
Age (yrs)	73 (61–81)
Age (yrs) at KB1	59 (54–66)
Age (yrs) at KB2	65 (55.5–72)
Gender	
Female	16 (55%)
Male	13 (45%)
Race	
Caucasian	24 (83%)
African American	4 (14%)
Others	1 (3%)
Clinical diagnosis	
GPA	10 (34%)
MPA	19 (66%)
Organ involvement	
Kidney	29 (100%)
Lung	8 (28%)
Sinus	6 (21%)
Skin	5 (17%)
Joint	3 (10%)
Ear, nose and throat	2 (7%)
Nerve	1 (3%)
Indications for KB2	
Elevation in serum creatinine	16 (55%)
Proteinuria	14 (48%)
Persistent hematuria	10 (34%)
New hematuria	10 (34%)
Positive ANCA	1 (3%)
Active ANCA glomerulonephritis in KB2	11 (38%)
ANCA type at KB1	
Proteinase-3-ANCA	9 (31%)
Myeloperoxidase-ANCA	17 (58%)
Negative-ANCA	3 (10%)
ANCA status at KB2	
Newly positive	9 (31%)
Persistently positive	8 (28%)
Unknown	6 (21%)
Negative	5 (17%)
Serum creatinine (mg/dl) at KB1	2 (1.6-4.45)
Serum creatinine (mg/dl) at KB2	1.9 (1.2-3.8)
Histopathological class in KB1 (*n* = 29)	
Focal	8 (28%)
Crescentic	5 (17%)
Mixed	13 (45%)
Sclerotic	3 (10%)
Renal Risk Score in KB1 (n=29)	
Low	8 (28%)
Medium	17 (59%)
High	4 (14%)
Histopathological class in KB1 in patients with active ANCA-GN in KB2 (*n* = 11)	
Focal	3 (27%)
Crescentic	1 (9%)
Mixed	7 (64%)
Sclerotic	0
Histopathological class in KB2 in patients with active ANCA-GN in KB2 (*n* = 11)	
Focal	3 (27%)
Crescentic	0
Mixed	5 (45%)
Sclerotic	3 (27%)
Renal Risk Score in KB1 in patients with active ANCA-GN in KB2 (*n* = 11)	
Low	5 (45%)
Medium	5 (45%)
High	1 (3%)
Renal Risk Score in KB2 in patients with active ANCA-GN in KB2 (*n* = 11)	
Low	3 (27%)
Medium	5 (45%)
High	3 (27%)
Induction treatment after KB1	
Glucocorticoids	29 (100%)
Cyclophosphamide	18 (62%)
Rituximab	10 (34%)
Mycophenolate Mofetil	1 (3%)

ANCA-GN, ANCA-glomerulonephritis; GPA, granulomatosis with polyangiitis; KB1, initial kidney biopsy; KB2, repeat for-cause kidney biopsy; MPA, microscopic polyangiitis.

Data are presented as number of patients (%) or median (interquartile range).

After KB2 was performed, 13 patients (45%) had a change in their immunosuppression plan: 11 with active disease requiring induction immunosuppression and 2 with inactive disease who were transitioned from induction to maintenance therapy. Progression to end-stage kidney disease occurred in 12 (41%) patients, of which 5 patients had active disease on KB2. In those with active ANCA-GN in KB2 (*n* = 11), 45% developed end-stage kidney disease.

**Figure 1:**
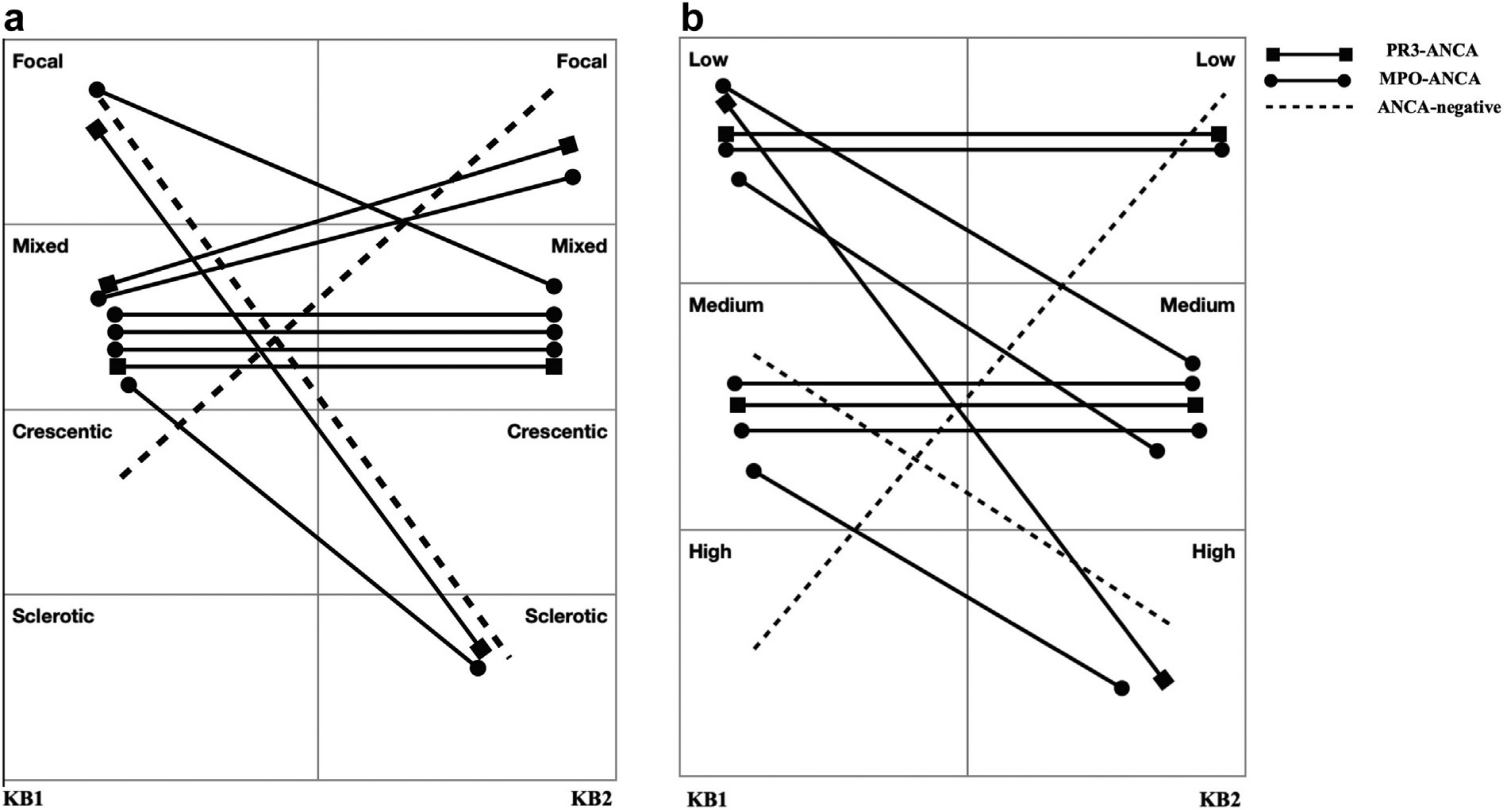
ANCA type, histopathological ANCA-GN Class, renal risk score (RRS) in KB1 and KB2 in patients with active ANCA-GN in KB2 (*n* = 11). (a) ANCA-GN histopathological class in KB1 and KB2. (b) Renal Risk Score in KB1 and KB2. ANCA-GN, ANCA-glomerulonephritis; KB1, initial kidney biopsy; KB2, repeat for-cause kidney biopsy; MPO, myeloperoxidase; PR3, proteinase-3. ^a^Based on criteria used by the International Working Group of Renal PathologistsS3 Renal Risk Score.S4

We analyzed clinical and histopathologic parameters in the 11 (37%) patients with active ANCA-GN in KB2. The indications for KB2 in this cohort were serum creatinine elevation (82%), proteinuria (36%), new hematuria (54%), and persistent hematuria (27%). A mixed class ANCA-GN was the most common class in KB1 (*n* = 7) and KB2 (*n* = 5). Sclerotic class ANCA-GN was absent in KB1, whereas a crescentic class was absent in KB2. Of the 3 patients with focal class ANCA-GN in KB1, 1 had mixed class whereas the remaining 2 had sclerotic class ANCA-GN in KB2. Among the 7 patients who had mixed class ANCA-GN in KB1, 4 remained as mixed class ANCA-GN, 2 had focal class ANCA-GN, and 2 had sclerotic class ANCA-GN in KB2. The most common renal risk score (RRS) was low and medium in KB1 (*n* = 5 equally). In the low risk group, 2 patients progressed to medium risk and 1 patient progressed to severe risk in KB2. In the medium risk group, 2 patients progressed to severe risk in KB2 (Figure 1a and b).

The percentage of global sclerosis increased from 20% to 43%, and cellular crescents decreased from 14% to 0% in KB2 (*P* = 0.01 and *P* = 0.02, respectively). The percentage of fibrous crescents was unchanged (*P* = 0.81), and a trend in decreased fibrinoid necrosis and increased segmental sclerosis was noted in KB2. Severe interstitial fibrosis and tubular atrophy, absent in KB1, was present in 3 patients in KB2.

## Discussion

This study aimed to evaluate the indications, clinical, and histological parameters in patients with ANCA-GN who underwent a repeat kidney biopsy. It shows that 38% of patients had active ANCA-GN in KB2. Among patients with active GN, elevated serum creatinine was the most common reason for a repeat kidney biopsy. Active GN was present in KB2 in 70% of patients with new onset hematuria and 20% of patients with persistent hematuria. The risk of end-stage kidney disease is high in patients with active disease in KB2.

Chapman *et al.*^6^ reviewed 59 interval biopsies to assess response to induction therapy and found active disease in 42% of cases on repeat kidney biopsy, which led to a change in immunosuppressive plan in 75% of patients. Our study shows a similar percentage of active ANCA-GN on repeat biopsies (38%), but a lower rate of immunosuppression change (45%). This may be due to differences in physician practices, the presence of extrarenal manifestations, and differences in pathology found in KB2. Similar to our study, the majority of patients in the Chapman *et al.*^6^ study received cyclophosphamide followed by rituximab. However, it is worth noting that none of our patients received plasma exchange, compared to 47% of patients in the Chapman cohort.

The data on the value of persistent hematuria is conflicting. One study concluded that despite clinical remission, persistent hematuria, but not proteinuria, predicted future renal relapses. Interestingly, persistent hematuria did not correlate with end-stage kidney disease.^2^ On the other hand, a recent study noted a correlation between proteinuria and hematuria at the end of induction therapy with future relapses, renal failure, and death.^1^ In our study, only 20% of patients with persistent hematuria had active GN. In addition, although proteinuria was a major indication for repeat kidney biopsy, it presented a small percentage of patients with active GN. This is consistent with our current understanding that proteinuria is a marker of damage rather than active GN, although the data on its prognostic value seems to differ depending on the study looked at.^1^^,^^2^ Our study suggests that in patients with persistent hematuria and proteinuria, instead of preemptive escalation of immunosuppression, a repeat kidney biopsy should be considered to guide immunosuppression.

The finding of increased chronicity with higher percentages of global sclerosis and interstitial fibrosis and tubular atrophy in KB2 has also been noted in a previous study that looked at protocolized kidney biopsies in ANCA-GN.^7^ In terms of class switching, 63% of our patients class-switched, and the remaining (36%) had unchanged ANCA-GN class. Among those who class-switched, 36% progressed and 27% improved. These numbers are similar to the Chapman study.^6^ In addition, our study shows that patients with focal class GN progressed to either mixed or sclerotic class which has a less favorable outcome. This could be a reflection of sampling error or differences in disease characteristics and treatment response.

Our study looked at the RRS initially published by Brix *et al.*S4 In our study, Brix RRS was unchanged in 45%, improved in 10%, and worsened in 45% of patients, again suggesting a higher degree of chronicity in KB2. This is in contrast to the study by Chapman *et al.*^6^ where 30% showed improvement in Brix RRS whereas 20% showed progression to higher Brix RRS. These differences could be due to differences in patient demographics and practice patterns.

Although limited by the small sample size, the data of this study support histologic confirmation of disease activity rather than relying on current conventional markers to improve prognostication and guide treatment decisions. Larger studies are needed to confirm the findings of this study.

## Disclosure

DG reports being a consultant to ChemoCentryx, GSK, Otsuka, Calliditas, Amgen, and Aurinia Inc. FA and LX declare no conflicting interests.

## Supplementary Material

**Supplementary Methods.**

**Supplementary Reference.**

**Supplementary Table S1.** Details of KB2 indication.
