# Supplementary material for: Repeat Kidney Biopsies in Anti-Neutrophil Cytoplasmic Autoantibody-Associated Vasculitis: Clinical and Histologic Progression
Source: Kidney Int Rep. 2023 Jul 29;8(10):2146–9. doi: 10.1016/j.ekir.2023.07.021 (PMC10577318; doi:10.1016/j.ekir.2023.07.021)
Supplement: Supplementary File (PDF) [file mmc1.pdf]

Repeat Kidney Biopsies in ANCA-associated Vasculitis: Clinical and Histologic Progression

Supplementary Material

Supplementary Methods

Supplementary References

Table S1. Details of KB2 indication

Supplementary Methods

We conducted a single-center retrospective analysis of patients diagnosed with ANCA-GN on an initial kidney biopsy (KB1) who had a for-cause repeat kidney biopsy (KB2). AAV patients diagnosed between January 1995 - April 2022 were identified from the Johns Hopkins Vasculitis Center database. To be included in the study, patients needed to be above the age of 18 years old with an initial kidney biopsy confirming pauci-immune ANCA glomerulonephritis (ANCA-GN). Additionally, patients needed to have a for-cause repeat kidney biopsy at any point during their disease course until the time of last follow-up. The reason to perform a kidney biopsy was left at the discretion of the treating provider. Patients < 18 years old and those with concomitant anti-glomerular basement membrane (anti-GBM) disease were excluded. Data were collected retrospectively using electronic medical health records. Baseline characteristics, histopathological parameters and ANCA GN class, Renal Risk Score (RRS), and outcomes such as end-stage kidney disease (ESKD) and death were recorded.

Baseline characteristics included age, gender, ANCA type, and clinical diagnosis. Clinical diagnosis of ANCA-associated vasculitis (AAV) was defined as granulomatosis with polyangiitis (GPA) or microscopic polyangiitis (MPO) based on the 2012 Chapel Hill Consensus Conference definitions for ANCA-associated vasculitis [S1]. Proteinuria was defined as greater than +1 protein on a urinalysis or a urine protein to creatinine ratio greater than or equal to 300 mg/g. Hematuria was defined as greater than 5 red blood cells per high power field on a urinalysis. Persistent hematuria was defined as the presence of greater than 5 red blood cells per high power field on a urinalysis after clinical remission was achieved otherwise. Active disease on KB2 was defined as crescentic pauci-immune glomerulonephritis. Inactive disease on KB2 was defined as a diagnosis other than crescentic pauci-immune glomerulonephritis. The estimated glomerular filtration rate (eGFR) was calculated using the Chronic Kidney Disease Epidemiology Collaboration (CKD-EPI) equation [S2]. Renal histopathology was grouped into four classes based on criteria used by the International Working Group of Renal Pathologists: Focal (≥50 % normal glomeruli), Crescentic (≥50 % glomeruli with cellular crescents), Mixed (<50 % normal, <50 % crescentic, <50 % globally sclerotic glomeruli), and Sclerotic (≥50 % globally sclerotic glomeruli) [S3]. Renal Risk Score was calculated to predict the risk of ESKD at 36 months and patients were classified as low risk (points 0), medium risk (points 2-7), high risk (points 8-11) in accordance with the 2018 Renal Risk Score by Brix and colleagues [S4]. Interstitial fibrosis and tubular atrophy (IFTA) was classified as none (0%), mild (1-25%), moderate (26-50), and severe (greater than 50%).

Descriptive statistics were used. Continuous measures were reported as mean, standard deviation (SD), median, and interquartile range (IQR). Categorical measures were reported as count and percent. Significance was assessed via analysis by Wilcoxon matched-pairs signed rank test or McNemar's Chi-squared test as appropriate. A p-value ≤ 0.05 was considered statistically significant in all cases.

Supplementary References

S1. Jennette, J. C., Falk, R. J., Bacon, P. A., Basu, N., Cid, M. C., Ferrario, F., Flores-Suarez, L. F., Gross, W. L., Guillevin, L., Hagen, E. C., Hoffman, G. S., Jayne, D. R., Kallenberg, C. G., Lamprecht, P., Langford, C. A., Luqmani, R. A., Mahr, A. D., Matteson, E. L., Merkel, P. A., Ozen, S., … Watts, R. A. (2013). 2012 revised International Chapel Hill Consensus Conference Nomenclature of Vasculitides. Arthritis and rheumatism, 65(1), 1–11.

S2. Levey, A. S., Stevens, L. A., Schmid, C. H., Zhang, Y. L., Castro, A. F., 3rd, Feldman, H. I., Kusek, J. W., Eggers, P., Van Lente, F., Greene, T., Coresh, J., & CKD-EPI (Chronic Kidney Disease Epidemiology Collaboration) (2009). A new equation to estimate glomerular filtration rate. Annals of internal medicine, 150(9), 604–612. https://doi.org/10.7326/0003-4819-150-9-200905050-00006

S3: Berden, A. E., Ferrario, F., Hagen, E. C., Jayne, D. R., Jennette, J. C., Joh, K., Neumann, I., Noël, L. H., Pusey, C. D., Waldherr, R., Bruijn, J. A., & Bajema, I. M. (2010). Histopathologic classification of ANCA-associated glomerulonephritis. Journal of the American Society of Nephrology : JASN, 21(10), 1628–1636. https://doi.org/10.1681/ASN.2010050477

S4. Brix SR, Noriega M, Tennstedt P, et al. Development and validation of a renal risk score in ANCA-associated glomerulonephritis. Kidney Int. 2018;94(6):1177-1188. doi:10.1016/j.kint.2018.07.020

Table S1. Details of KB2 indication (n=29)

Indication	
**Elevation in serum creatinine only**	5 (17%)
**Proteinuria only**	3 (10%)
**New hematuria only**	2 (7%)
**Persistent hematuria only**	2 (7%)
**Elevation in serum creatinine + new hematuria + proteinuria**	4 (14%)
**Elevation in serum creatinine + persistent hematuria + proteinuria**	1 (3%)
**Elevation in serum creatinine + new hematuria**	3 (10%)
**Elevation in serum creatinine + proteinuria**	1 (3%)
**Elevation in serum creatinine + persistent hematuria**	2 (7%)

Indication	
**Peristent hematuria + proteinuria**	4 (14%)
**Persistent hematuria + positive ANCA**	1 (3%)
**New hematuria + proteinuria**	1 (3%)

KB2, repeat for-cause kidney biopsy
